# Exploring hierarchical and overlapping modular structure in the yeast protein interaction network

**DOI:** 10.1186/1471-2164-11-S4-S17

**Published:** 2010-12-02

**Authors:** Changning Liu, Jing Li, Yi Zhao

**Affiliations:** 1 Bioinformatics Group, Key Laboratory of Intelligent Information Processing, Center for Advanced Computing Research, Institute of Computing Technology, Chinese Academy of Sciences, Beijing, PR China

## Abstract

**Background:**

Developing effective strategies to reveal modular structures in protein interaction networks is crucial for better understanding of molecular mechanisms of underlying biological processes. In this paper, we propose a new density-based algorithm (ADHOC) for clustering vertices of a protein interaction network using a novel subgraph density measurement.

**Results:**

By statistically evaluating several independent criteria, we found that ADHOC could significantly improve the outcome as compared with five previously reported density-dependent methods. We further applied ADHOC to investigate the hierarchical and overlapping modular structure in the yeast PPI network. Our method could effectively detect both protein modules and the overlaps between them, and thus greatly promote the precise prediction of protein functions. Moreover, by further assaying the intermodule layer of the yeast PPI network, we classified hubs into two types, module hubs and inter-module hubs. Each type presents distinct characteristics both in network topology and biological functions, which could conduce to the better understanding of relationship between network architecture and biological implications.

**Conclusions:**

Our proposed algorithm based on the novel subgraph density measurement makes it possible to more precisely detect hierarchical and overlapping modular structures in protein interaction networks. In addition, our method also shows a strong robustness against the noise in network, which is quite critical for analyzing such a high noise network.

## Background

Proteins, as the important players of cell machinery, often cooperate with other functional correlates to form protein complexes or functional modules when performing certain biological activities. Therefore, revealing modular structures in biological networks would help us to develop more effective protein function prediction algorithms and get a better understanding of molecular mechanisms of biological processes [1]. Recent progress in the proteomics technologies has enabled scientists to identify protein interactions on a genomic scale [2,3]. Such data can be generally modeled as a graph in which vertices represent proteins and edges represent interactions between them. Hence, it is not surprising that a variety of graph-theory approaches had been applied to analysis of protein-protein interaction (PPI) networks. So far, these analyses have revealed a number of distinctive topological properties, including power-law degree distribution, small world and high clustering coefficients [4]. However, uncovering the modular structures, as one of the key points, remains a non-trivial task due to several handicaps.

First, there is only very limited overlapping among existing high-throughput PPI datasets, which indicates that many of the detected interactions might be false positives [5]. These non-negligible uncertainties of protein interaction data lead to huge challenges to classical graph partitioning/clustering methods, despite their remarkable achievements in other fields. The specific topological characteristic of PPI network is another important cause [4]. As a scale-free network, PPI network's node connectivity distribution follows a power law, with a few nodes of highly connection and most others of low degree. Consequently, the results of traditional module detection methods usually failed into a dilemma -- finding either a handful of giant clusters or a great number of tiny cliques. Furthermore, the fact that PPI network is always organized into a complicated hierarchical and overlapping modular structure makes it even harder to develop a competent method, as to accurately extract nested modules with possible overlaps [4].

Recently, a number of network clustering algorithms introduced by different research groups in diverse fields, have been applied to identify functional modules from complex PPI networks. Considering the special role of hubs in a scale-free network, Cho and Zhang restructured a complex interactome network into a hub-oriented hierarchical tree based on the path strength model, and then identified structural hubs and functional modules on the basis of hub confidence scores [6]. Using gene expression data as an additional input to assess the quality of interactions, Chin et al. developed a novel hub-attachment based agglomerative clustering method to detect functional modules from confidence-scored protein interactions and expression profiles [7]. In contrast to the traditional concept of a module as a group of cohesively interacting proteins, Pinkert et al. presented an alternative module finding approach of decomposing a network into functional roles, which based on a self-consistent definition independent of any prior knowledge of functional modules [8].

Density-based clustering algorithms, which search for highly-connected regions within a network, have been proved to be fairly effective in identifying meaningful clusters from datasets with high-level noise [9]. For detecting functional modules in PPI networks, a number of density-based clustering algorithms have been recently proposed. These methods often vary in the means used to assess the density of the subgraphs. The most stringent criterion is used by Maximal Clique algorithm, which identify fully-connected subgraphs, k-cliques, in the network [10]. In comparison, other algorithms exploited the relatively more comprehensive criteria: MCODE applied the network partitioning based on the density of k-cores [11]; CFinder interpreted network modules as unions of all adjacent k-cliques [12]; DPClus identified subgraphs that satisfied certain cluster density and connectivity properties [13]. Although there were some progress, how to properly assess the density of the subgraphs is not quite settled yet.

In this paper, we put forward an effective and efficient network clustering algorithm named ADHOC (A Density-based Hierarchical and Overlapping Clustering method) based on a novel subgraph density measurement. By statistically evaluating several independent criteria, we found that our method could significantly improve the outcome as compared with previously reported density-based methods. Next, we applied ADHOC to investigate the hierarchical and overlapping modular structure in the yeast PPI network. As shown in the results, our method can effectively detect both protein modules and the overlaps between them, which would greatly promote the precise prediction of protein functions. Moreover, by further assaying the intermodule layer of the yeast PPI network, we classified two types of protein hubs, module hubs and inter-module hubs. Each group of hubs presented distinct characteristics both in network topology and biological functions, which could assist us to better understanding of the relationship between network architecture and biological implications.

## Methods

### The principle of ADHOC method

Since PPI network is a very noisy environment, here we constructed our clustering method ground on the idea of density-based clustering. As a popular metric of graph theory, the clustering coefficient -- a real number ranging from 0.0 to 1.0, is a reasonable measurement that can reflect the local-density of a node's neighborhood. However, as the connectivities of different nodes in PPI network vary significantly, it is not rational to use a fixed threshold of the clustering coefficient, which determines whether a node's neighborhood is a density region. For a node of degree 2, the value of its clustering coefficient equals to 1.0, which means the node and its neighbors form a triangle, is fairly common in PPI network. On the other hand, to detect a node of degree 50 with the clustering coefficient equals to 1.0 seems impossible. Hence different from traditional density-based clustering metods, by adding node degree as an important parameter into our model, we proposed a novel subgraph density measurement approach (Formula 1) which would assign different thresholds to nodes with different degrees according to the presetting density levels.(1)

In this model, the clustering coefficient threshold, MinCC, for a given node is decided by two parameters: first is its degree d, second is the size k of the clique with which it compared. When a node's clustering coefficient value is no less than its MinCC, we reckon that this node's neighborhood is a density region which is at least as dense as the k-clique it compared with. The MinCC is computed by comparing the p-value of detecting no less than C edges in the node's neighborhood by chance alone, given by the cumulative probability of binomial distribution, with the p-value of finding a k-clique in the PPI network by chance. Notably, as the PPI networks present a trait of clustering, that is, two vertices that are both neighbors of the same third vertex have a heightened probability of also being neighbors of one another, the probability p of detecting an edge by chance in the Formula 1 is defined as the global clustering coefficient. In addition, although the range of the clique size k is usually positive integers, in Formula 1 we can extend the range of k to any positive real numbers larger than one, which would be useful to precisely detect density regions in PPI networks.

**Figure 1:**
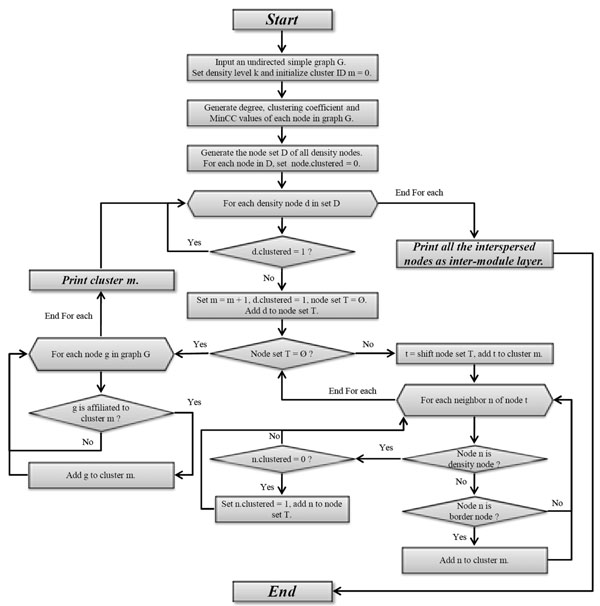
**Flowchart of the proposed algorithm** For a given density level k, all nodes are classified into four types, and then clustered into different modules or inter-module layer according to their types.

By using the above model, for a given k, all the nodes in the PPI network can be classified as four types. First is "density node" whose clustering coefficient value is no less than the threshold given by MinCC. The density region of one density node is defined as its immediately connected neighbors, except those without any connected edges to any other neighbors. Second is "border node" which is not a density node but still in the density region of a density node. A border node could be in the density regions of different density nodes at the same time. Third is "affiliated node", a node is an affiliated node to one cluster if all its edges are connected to the nodes in this cluster. All the remaining nodes are classified as "interspersed node", which is most likely to be the noise in the network, or the intermediate that connect clusters with each other. Based on the node classification, the core clustering method basically consists of following steps (the detailed flowchart of the overall algorithm is illustrated in Figure 1): 1) for a given k, classify all nodes to four types; 2) for any two density nodes, if they are directly connected, they are put into the same cluster; 3) border nodes are put into the same clusters as their directly connected density nodes; 4) all the affiliated nodes will be placed to the clusters which they are affiliated with; 5) all the interspersed nodes are grouped to inter-module layer which don't fall into any clusters.

**Figure 2:**
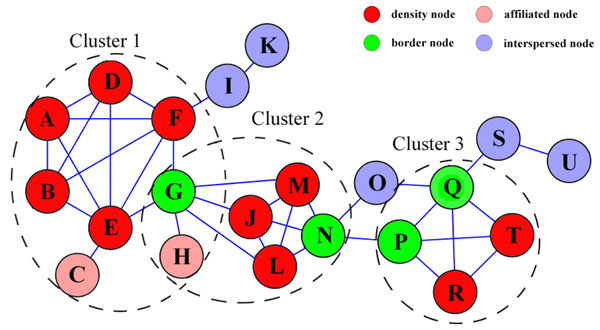
**A simple demonstration of ADHOC method** The four type's nodes are marked by different colors. The identified three clusters are circled by dashed lines.

As a demonstration, the result from the analysis of a simple network was presented in Figure 2. Given k = 3, this simple network, composed of 21 nodes and 36 edges in total, was sorted into 3 clusters, with five remaining nodes marked as interspersed nodes. It is worth noting that, in the clustering process, different clusters could be overlapped because both border nodes and affiliated nodes can be sorted into multiple clusters simultaneously, such as node G and H in Figure 2.

### Data sources

The yeast (Saccharomyces cerevisiae) protein interaction dataset that we used is the core interaction data from the DIP database (dip.doe-mbi.ucla.edu, date 2007-10-07). This dataset contains 2779 distinct proteins and 6212 filtered reliable interactions (self-interactions were discarded).

The Gene Ontology (GO) data are obtained from the SGD database (www.yeastgenome.org, the GO-Slim data, date 2008-06-07), which contained 12,628 cellular component terms, 8,199 molecular function terms and 13,356 biological process terms.

The lethality and the phenotype data for the yeast protein interaction dataset are obtained from the MIPS database (mips.helmholtz-muenchen.de, date 2006-05-18). The lethality dataset lists whether yeast strains are viable or not when the specific genes are knockout. The phenotype dataset is a list of phenotypes observed as the consequences of gene knockouts.

### Global clustering coefficient

The global clustering coefficient calculates the number of closed triplets (or 3 x triangles) proportional to the total number of connected triples (both open and closed). This measure was designed to give an overall indication of the clustering in the whole network.(2)

### Local clustering coefficient

The local clustering coefficient of a vertex in a graph quantifies how close its neighbors are to being a clique. For a vertex v with degree k_v_, the local clustering coefficient is defined as |E|/(k_v_(k_v_-1)/2), where |E| is the number of edges between the vertex's neighbors and k_v_(k_v_-1)/2 is the theoretical maximum number of edges possible.

### P-value of GO

The extent to which the clusters are associated with a specific GO term is evaluated using a p-value based on the hypergeometric distribution. Here N, n and M are the sizes of the whole network, a cluster and proteins which annotated with the specific GO term in the network respectively and m is the number of proteins annotated with the specific GO term in the cluster. In this paper, all p-values were corrected with Bonferroni correction for multiple hypothesis testing. Because the p-values are frequently small numbers with positive values between 0 and 1, the negative logarithms (to base 10, denoted -log p) are used.(3)

### P-value of lethality

The p-value that a group of proteins would be enriched with lethal proteins by chance alone is given by the cumulative probability of binomial distribution as(4)

Here n is the size of the protein group, k is the number of lethal proteins in the group, and p is the probability that a protein be lethal.

### Vertex between ness

Vertex betweenness, first proposed in social network research, has been studied in the past as a measure of the centrality and influence of nodes in networks. The betweenness centrality of a vertex i is defined as(5)

Here *δ_s,t_* is the number of geodesies linking nodes s and t, and *δ_s,t_* (*i*) is the number of geodesies linking nodes s and t that contain i.

## Results

### Evaluation of ADHOC: comparative assessment and robustness analysis

To demonstrate the strength of the ADHOC approach, we compared it with other five clustering algorithms which are also based on the idea of density region detection, including Maximal Clique, DPClus, IPCA, MCODE and Cfinder [10-14]. For those algorithms based on k-clique finding, such as Maximal Clique or Cfinder, k is typically suggested to set to 4 [10,12]. Correspondingly, in ADHOC, the density level parameter k was assigned to 3, since it is used to measure the density of a node's neighborhood. For other three clustering algorithms, we used their default parameter settings: DPClus CP_in_ = 0.5, D_in_ = 0.9; IPCA T_in_ = 0.9, SP < 3; MCODE VWP = 0.1, Fluff = 0. The comparison results for the core interaction data from the DIP database are summarized in Table 1. The performance was measured by the node discard rate and the enrichment of the Gene Ontology categories (molecular functions, biological process, cellular component). The -log p-values in Table 1 are the average -log p-values of all detected clusters by each method.

**Table 1:** Comparison of ADHOC to Competing Clustering Methods for DIP Yeast PPI Dataset

Method	Cluster Number	Cluster Size	Discard (%)	GO (-log P-value)		

				MF	BP	CC
ADHOC	50	20.56	68.05	5.18	7.44	6.38
Maximal Clique	376	4.55	80.06	3.43	4.02	2.67
IPCA	253	4.64	80.39	3.61	4.09	2.89
DPClus	90	5.27	84.49	3.91	4.50	3.44
MCODE	29	23.76	75.21	4.20	5.23	4.89
CFinder	84	7.46	80.06	4.63	6.03	4.66

Cluster Number: the number of clusters identified by each method; Cluster Size: the average number of proteins in each cluster; Discard (%): the percentage of proteins not assigned to any cluster; GO: the average -log p-values (adjusted) of all detected clusters for Gene Ontology (molecular functions (MF), biological process (BP), and cellular component (CC)).

Table 1 shows that, compared with the other five methods, ADHOC has the least portion of proteins which are discarded to create clusters. On average, ADHOC collects 300 more proteins into the clusters than other five methods. Moreover, by the enrichment analysis of Gene Ontology categories, those clusters obtained by ADHOC showed a dominant superiority on all the categories over five others. Therein, the clusters on molecular functions category, have p-values that are approximately 4-fold, 10-fold, 20-fold, 30-fold and 60-fold lower than CFinder, MCODE, DPClus, IPCA and Maximal Clique method, respectively. On biological process category, the p-values of the clusters identified by ADHOC are approximately 25-fold, 150-fold, 800-fold, 2000-fold and 2500-fold lower than CFinder, MCODE, DPClus, IPCA and Maximal Clique method, respectively. While on cellular component category, the clusters detected by ADHOC possess p-values that are approximately 50-fold, 30-fold, 800-fold, 3000-fold and 5000-fold lower than CFinder, MCODE, DPClus, IPCA and Maximal Clique method, respectively.

As mentioned above, clustering algorithms for PPI network should be insensitive to noise due to the fact that PPI network often contains a huge amount of noise. We therefore examined the robustness of ADHOC to noises in the PPI network. The performance of ADHOC was evaluated by adding 5% ~ 25% random interactions to unconnected protein pairs in the PPI network. For each noise percentile, we generated 100 noise-added networks and then re-analysed. Table 2 summarizes the number of clusters detected by ADHOC and the corresponding average -log p-values for the Gene Ontology categories. The performance of ADHOC was found to be very robust to the addition of random interactions, even if the ratio of noise reached to 25%. The slight decreasing in the average number of detected clusters could be explained by the increased network connectivity.

**Table 2:** Robustness Analysis of ADHOC

Noise	Cluster Number	GO MF (-log P-value)	GO BP (-log P-value)	GO CC (-log P-value)
0%	50	5.18	7.44	6.38
5%	44.15 ± 2.28	5.19 ± 0.19	7.34 ± 0.31	6.52 ± 0.35
10%	42.49 ± 2.50	5.13 ± 0.28	7.26 ± 0.33	6.50 ± 0.35
15%	39.94 ± 2.69	5.20 ± 0.28	7.31 ± 0.44	6.51 ± 0.49
20%	37.43 ± 3.40	5.28 ± 0.48	7.54 ± 0.62	6.66 ± 0.53
25%	35.13 ± 2.98	5.29 ± 0.42	7.47 ± 0.54	6.59 ± 0.54

Noise column represents the percentile of random noise added into DIP Yeast PPI dataset. For each noise percentile, we generated 100 random networks. The numbers in each cell indicate the values of Mean and Standard Variance.

### The effect of k on clustering

As for density level parameter k, we can easily prove that, in the case of k_2_ > k_1_ > 0, any cluster obtained when k = k_2_ must be subordinate to a cluster of the ones when k = k_1_. Thus, changing the parameter k is like adjusting the resolution of a zoom lens: increasing k makes the detected density region smaller but also more cohesive. Therefore, we can obtain the hierarchical and overlapping modular structure of PPI network by recursively using a set of given k values (k_1_, k_2_, …, k_n_) that increase gradually.

**Figure 3:**
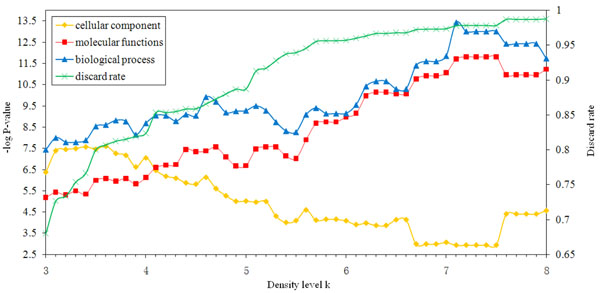
**The effect of k on clustering** The impact of different k value (ranging from 3 to 8 with a step of 0.1) on protein discard rate and Gene Ontology enrichment.

To finely investigate the effect of k value on network clustering, we set k ranging from 3 to 8 with a step of 0.1 and examined the impact of different k value on protein discard rate and Gene Ontology enrichment. As shown in Figure 3, the protein discard rate fluctuates in line with k value: as k = 3, about 68% nodes were discarded; as k = 5, 88% nodes were discarded; while k = 8, almost 98% nodes were discarded. On the other hand, the increase of k value has a relative complicated effect on Gene Ontology enrichment. For molecular function and biological process categories, both -log P-values rose along with k value: when k = 3, their -log P-values are 5.18 and 7.44 respectively; as k value further rose from 5 to 8, the -log P-values of the two simultaneously increased from 6.69 to 11.23, and 9.28 to 11.74, respectively. However, for cellular component category, its -log P-values showed the tendency of ascend first and then descent with the increase of k value: as k first rose from 3 to 3.5, its -log P-values increased from 6.38 to 7.47; but as k further increased to 5, the -log P-value failed to 5.02. Therefore, based on an overall consideration of the effects that different k values put on protein discard rate and Gene Ontology enrichment, we suggest that using such a value of k between 3 and 5 is reasonable in typical PPI network analysis. For the optimal step-size, now that both protein discard rate and Gene Ontology enrichment have a relative smooth change against k value, we thought that setting step-size to 0.5 would be a rational selection.

### The hierarchical and overlapping modular structure of the yeast PPI network

**Figure 4:**
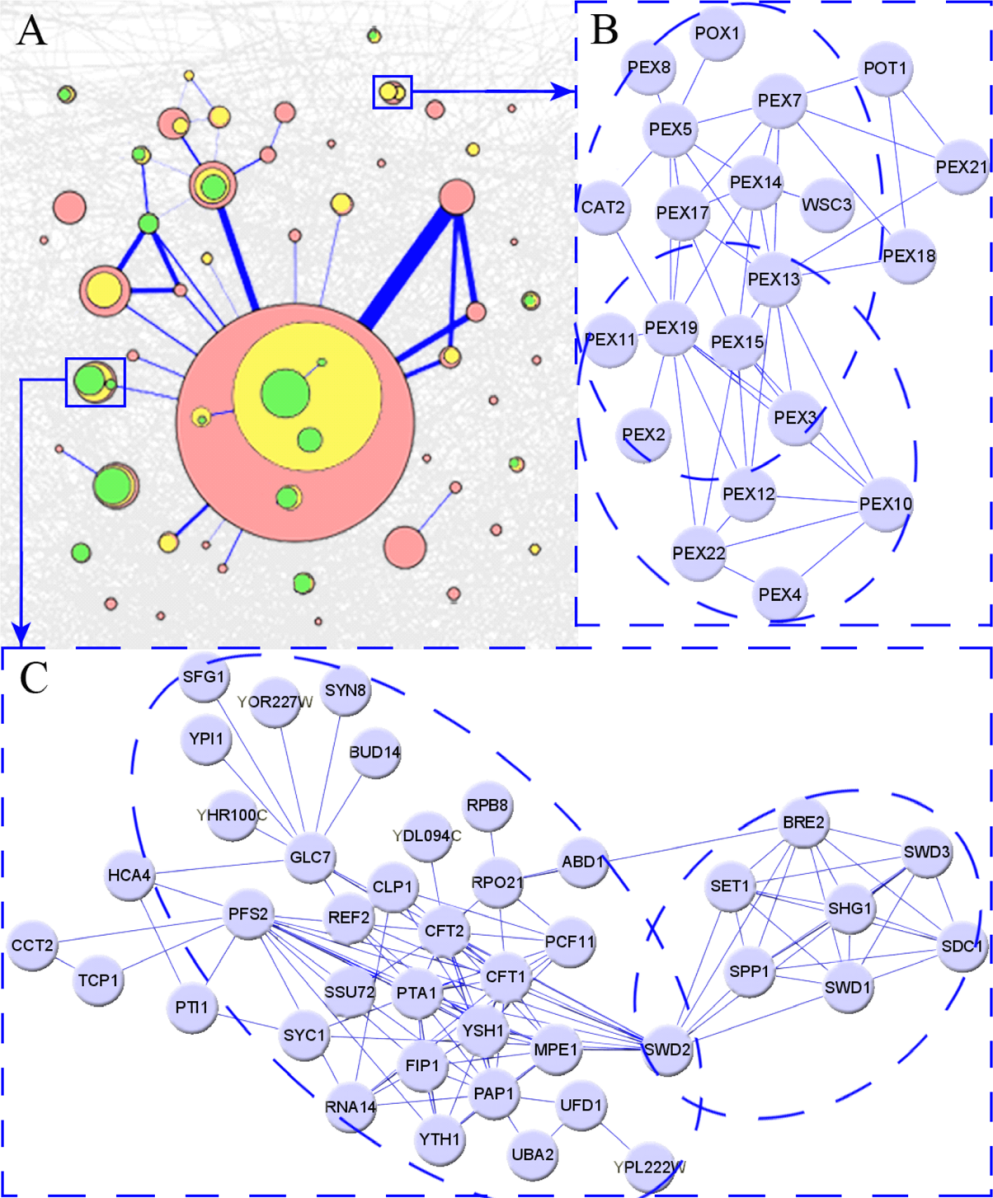
**The modular structure of the yeast PPI network** A) The hierarchical and overlapping modular structure of the yeast PPI network. B) The module of peroxisomal membrane proteins. C) The COMPASS complex and the CPF complex.

To investigate the hierarchical and overlapping modular structure in the yeast PPI network, we applied ADHOC to the core data of yeast PPI network using a set of k values (k = 3, 3.5, 5). By representing the modules obtained with different k values by nodes of different colors, and the overlapping between the modules by edges linking the nodes, we can naturally depict the complex modular structure of the yeast PPI network as a graph, as shown in Figure 4A. In this graph, the area of nodes and the width of edges are proportional to the size of the corresponding modules and to the size of the overlaps, respectively. When k = 3, the network could be clustered to 50 modules varying different size from 4 to 253. When more stringent criteria (k = 3.5, 5) were used, some modules were further divided to smaller ones, and therefore formed a hierarchical structure. These modules and especially the overlapping between them which are not easily revealed through conventional approaches, are believed to be biologically meaningful and ready to be deeply analyzed by further biological experiments. Among them, we here presented two interesting examples (Figure 4B and Figure 4C).

The subgraph which enlarged in Figure 4B is a 21-member cluster detected when k = 3. According to gene function annotations from SGD database, most members in the cluster are peroxisomal membrane proteins which function in peroxisomal matrix protein import [15,16], such as Pex2p and Pexl0p. In addition, there are also some function related proteins which allocated to peroxisome, such as Poxlp and Potlp which function in fatty acid metabolism. When k = 3.5, the cluster is further divided to two overlapping sub-clusters which is consistent with the known facts from SGD database that the peroxisomal import machinery are composed of two subcomplexes. The first one is the docking subcomplex, which comprises Pexl4p, Pexl7p and Pexl3p. And the second one is the translocation subcomplex, which contains Pex2p, Pexl0p and Pexl2p. Moreover, the proteins which located in the overlapping region of these two sub-clusters also have important functions: Pex3p and Pexl9p are identified as proteins required for the proper localization and stability of peroxisomal membrane proteins; Pexl lp and Pexl5p are required for peroxisome biogenesis.

It is generally believed that inter-overlapping modules in the network should have functional relevance to some extent. However, we have found this kind of overlapping between modules is sometimes caused by the fact that some of its shared members are proteins with multiple functions. Figure 4C shows a 41-node cluster detected when k = 3. GO annotations from SGD has shown that members in the cluster are mainly involved in two different biological process categories -- "protein modification" and "transcription". When k changes to 5, this cluster has been further divided into two parts. Different from the results in Figure 4B, these two sub-clusters neither are the subunits of some complex, nor have functional relevance. Therein, Shglp, Sdclp, Swdlp, Swd2p, Swd3p, Spplp, Bre2p, and Setlp make up the conserved COMPASS complex, which catalyzes methylation of histone H3 [17]. While other proteins, such as PAP1, YSH1, SSU72 and CFT1, compose CPF complex, which is a multisubunit complex that involved in RNAP II transcription termination [18]. These two complexes of different functions are connected by their shared protein Swd2p, which has dual functions in RNA polymerase II transcription termination and lysine 4 methylation of histone H3 [19].

Besides these cases in line with the known findings, there remain a lot of function unknown proteins in our modules. These modules that are obtained by clustering often correspond to some complexes and pathways, and enrich one or more functions in GO analysis. Therefore, the hierarchical and overlapping modular structure may promote the function prediction of unknown proteins in these modules. According to the results of GO enrichment analysis, we predicted the GO biological process of unknown proteins in various modules, as shown in Table 3. When a very stringent threshold (-log p-value > 10) was used, we predicted 58 proteins functions, which mainly focused on "transcription", "protein catabolic process" and "ribosome biogenesis and assembly".

**Table 3:** Prediction for uncharacterized proteins (ordered by predicted functions)

Protein	P-value	Predicted Function	Protein	P-value	Predicted Function
Q12156	12.28	cytoskeleton organization and biogenesis	P16387	19.64	transcription
Q05911	14.20	nuclear organization and biogenesis	P16547	19.64	transcription
P01097	19.56	precursor metabolites and energy generation	P25659	19.64	transcription
O13563	25.25	protein catabolic process	P36139	19.64	transcription
P36003	25.25	protein catabolic process	P38301	19.64	transcription
P50086	25.25	protein catabolic process	P38352	19.64	transcription
P53196	25.25	protein catabolic process	P38717	19.64	transcription
Q06665	25.25	protein catabolic process	P38915	19.64	transcription
Q05778	25.25	protein catabolic process	P39113	19.64	transcription
P39713	19.31	protein catabolic process	P39533	19.64	transcription
P42942	19.31	protein catabolic process	P40560	19.64	transcription
P53243	19.31	protein catabolic process	P46954	19.64	transcription
P53743	19.31	protein catabolic process	P47005	19.64	transcription
P53851	19.31	protein catabolic process	P47120	19.64	transcription
Q03935	19.31	protein catabolic process	P53116	19.64	transcription
Q06512	19.31	protein catabolic process	P53878	19.64	transcription
Q08018	19.31	protein catabolic process	Q03899	19.64	transcription
P53724	14.63	protein catabolic process	Q04847	19.64	transcription
P40462	14.08	ribosome biogenesis and assembly	Q05947	19.64	transcription
P43584	14.08	ribosome biogenesis and assembly	Q06479	19.64	transcription
P47019	14.08	ribosome biogenesis and assembly	Q06640	19.64	transcription
P53163	14.08	ribosome biogenesis and assembly	Q07844	19.64	transcription
Q02608	14.08	ribosome biogenesis and assembly	Q08923	19.64	transcription
Q03162	14.08	ribosome biogenesis and assembly	Q12395	19.64	transcription
P38254	13.03	RNA metabolic process	Q12443	19.64	transcription
P38768	13.03	RNA metabolic process	P38182	23.45	vesicle-mediated transport
P53094	13.03	RNA metabolic process	Q12125	23.45	vesicle-mediated transport
P53212	13.03	RNA metabolic process	Q04562	20.50	vesicle-mediated transport
P53952	13.03	RNA metabolic process	Q12327	20.50	vesicle-mediated transport

The Swiss-Prot ID of proteins is listed in the Protein column, corresponding P-value (-log p-value > 10) is listed in the P-value column and predicted function for each protein is listed in the Predicted function column.

### Two types of hubs: module hubs and inter-module hubs

Different from other clustering methods, such as hierarchical clustering and k-means, the density clustering methods do not cluster all the nodes in PPI network. For those nodes that have been discarded in the density clustering, they are generally regarded as the noise in the network. Since these nodes are numerous in the network and many of them are network hubs, it might not be appropriate to just annotate all these nodes as noise and discard them. We speculate that these nodes spreading among the modules are likely to exercise different functions as compared with those within the modules. In order to reduce the impact of noise on our analysis, here we merely analyzed the hubs in PPI network. Yeast PPI network contains 261 hubs (degree > 10) in total. According to their location in the modules (when k = 3), these hubs can be divided into two categories: module hubs (192 nodes) and inter-module hubs (69 nodes). We then compared the topological characteristics and biological functions of these two types of hubs respectively.

**Figure 5:**
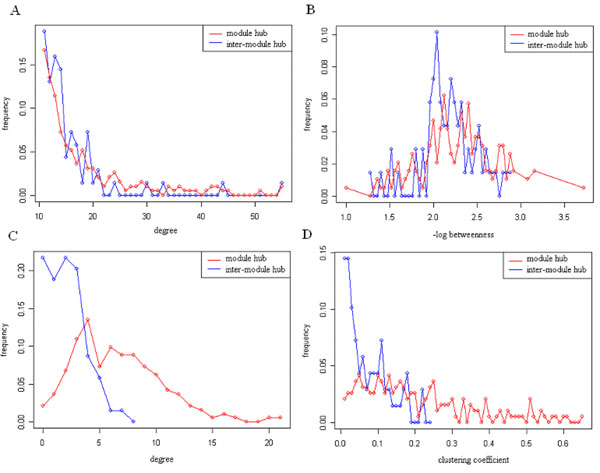
**The topological characteristics of module hubs and inter-module hubs** A) the degree distribution, B) the betweeness distribution, C) the interactions between hub nodes, D) the clustering coefficient distribution.

According to the topological characteristics of these two types of hubs, neither the degree distribution (Figure 5A) nor the betweeness value distribution (Figure 5B) has significant differences (Kolmogorov-Smirnov test, p-value > 0.05). However, if we only consider the interactions between hub nodes in each group (Figure 5C), the average number of the interactions between module hubs (6.54 ± 3.86) is obviously much greater than that between inter-module hubs (2.06 ± 1.66), indicating that module hubs are more prone to interact between themselves. The clustering coefficient distributions of these two groups (Figure 5D) also show the similar tendency. The average clustering coefficient of the module hubs (0.20 ± 0.15) is obviously much larger than the clustering coefficient of the inter-module hubs (0.07 ± 0.06), which means that the neighbours of the module hubs are more prone to interact with themselves.

Moreover, the GO enrichment analyses show that these two sets of hubs are markedly different in biological processes, molecular functions and cellular localizations (Table 4). In the biological process, module hubs significantly centralize on "Protein catabolic process", "RNA metabolic process" and "Nuclear organization and biogenesis" respectively, while inter-module hubs on "signal transduction", "anatomical structure morphogenesis" and "cell budding". On the molecular function, the top three functions of module hubs principally focus on "RNA binding", "peptidase activity" and "structural molecule activity", while inter-module hubs on "protein kinase activity", "signal transducer activity" and "DNA binding". In the cell location, module hubs are primarily at "nucleus", "endomembrane system" and "golgi apparatus", while inter-module hubs at "cell cortex", "site of polarized growth" and "cytoskeleton". In addition, the significant functional difference of two hubs is also reflected on their fatal (Table 5). Based on Yeast lethal gene data in MIPS database, we separately calculated the p-values of enriched lethal genes in all interspersed nodes, all module nodes, all hubs, inter-module hubs and module hubs. Compared with common nodes, the nodes within the hubs significantly enrich lethal genes (-log p-value = 7.09). However, when considering inter-module hubs and module hubs separately, lethal gene only enriches in the module hubs (-log p-value = 10.26), with no any accumulation in the inter-module hubs (-log p-value = 0.10), demonstrating the great contrast between fatal of two hubs.

**Table 4:** GO annotation (Top3) for module hubs and inter-module hubs

GO	Module Hubs		Inter-module Hubs	
**BP**	Protein catabolic process	7.64	Signal transduction	4.24
	RNA metabolic process	5.86	Anatomical structure morphogenesis	3.20
	Nuclear organization and biogenesis	3.95	Cell budding	3.08
**MF**	RNA binding	4.12	Protein kinase activity	3.26
	Peptidase activity	4.10	Signal transducer activity	2.33
	Structural molecule activity	1.48	DNA binding	1.45
**CC**	Nucleus	7.29	Cell cortex	1.69
	Endomembrane system	3.78	Site of polarized growth	1.46
	Golgi apparatus	2.71	Cytoskeleton	1.32

The numbers indicate the corresponding -log P-values.

**Table 5:** The enrichment of lethal genes in different groups

Type	Lethal	Viable	Unknown	Lethal %	-log P-value
**All Hubs**	119	131	11	45.59	7.09
**Module Hubs**	101	89	2	52.60	10.26
**Inter-module Hubs**	18	42	9	26.09	0.10
**Module Nodes**	210	397	89	30.17	0.33
**Interspersed Nodes**	386	1109	326	21.20	0.00

## Discussion and conclusions

Till now, a number of different assessment strategies, such as k-core, k-plex, k-block and n-clan, have been proposed to assess the density of highly connected regions. However, as the node connectivity distribution of PPI network follows a power law, generating clusters merely based on a fixed threshold of n or k is not rational. In this paper, by adding node degree as an important parameter, we developed a novel subgraph density measurement model which would assign different thresholds to nodes with different degrees according to the presetting density level parameter k. For ADHOC, the parameter setting is easy to use. As described above, for most PPI network, setting k in the range of 3~5 and step-size to 0.5 will meet the most requirements. When a small k is used in clustering, more proteins will be included into the modules, which in turn means more function-unknown proteins might be assigned to the functions of modules. Whereas, the average size of extracted modules using a small k is likely to be somewhat large, as a result more function terms might be enriched in each module by GO analysis. On the other hand, when using a big k in clustering, more proteins will be discarded so as to engender some smaller but more cohesive modules, which means less and more reliable novel protein function annotations. Therefore, for those clustering results based on ADHOC, we should integrate the relavent GO annotation of modules to further determine the function for those modules and their relationships with substructure.

With the progress of high throughput experimental techniques (e.g. yeast-two-hybrid), large-scale sets of protein interaction data are now publicly available for further bioinformatics studies. Based on the idea that proteins of similar functions tend to congregate into same modules, we can predict the functions of unknown proteins with high confidence according to extracted modular structures from PPI network. However, the functional analysis just using PPI has a limitation in accuracy because of its high-level noise of false positive and false negative interactions. Thus, how to construct a high reliable protein function prediction framework integrated with heterogeneous datasets (e.g. gene expression data, semantic knowledge) has been a challenge in the post-genomic era [7,20,21]. Moreover, exploring modular structures from protein interaction data can also enable us towards a better understanding of topological structures and the organizing principles of biological networks. For example, based on whether or not the hubs are co-expressed with their neighbors, Han et al. originally proposed a binary hub classification -- 'party hubs' and 'date hubs', and suggested that party hubs are local coordinators whereas date hubs are global connectors in the network [22]. Recently, by virtue of network motifs, Jin et al. further presented the concepts of 'motif party hubs' and 'motif date hubs', and showed that a network motif should be considered as an essential function unit in organizing modules of biological networks [23].

Previous cluster analysis on PPI network are mostly focused on prediction and analysis of network modules, but for those proteins that do not belong to any module, there was no much detailed study. In this work, by comparison of the topological characteristics and biological functions between module hubs and inter-module hubs, we speculate that inter-module hubs are more likely to play important roles in response to external signal stimulation and in coordinating the joint effect of many modules. Notablly, our prediction is in good agreement with the founding in breast cancer by Taylor et al., that is "Signaling domains were found more often in intermodular hub proteins "[24]. More interestingly, we found that there is significant divergence of fatality between inter-module hubs and module hubs. It is generally believed that the connections of a node in PPI network are closely related to its biological importance. Hence, hubs are more inclined to lethal gene [25]. However, some recent study found that this correlation might be worth exploring. For example, Zhang et al. suggested that the fatal tendency of hubs probably has no relationship with their impact on overall network topological features [26]. In this study, our results present a new possibility for this issue. We suggest that the definition of the existing hub is just a pure and simple topological concept. Hence, the interior of hubs should perform further division, while hubs in the different groups execute distinct important functions within biological networks.

For such a high noisy PPI network, density-based clustering method seems a very suitable choice to seek module structure. However, how to determine the module's density, as well as the density threshold, has no explicit standards. In order to solve this problem, in this paper, we developed a network clustering method (ADHOC) based on a novel density model. Using ADHOC, a PPI network could be divided into a hierarchical and overlapping modular structure. As compared with the existing density-dependent clustering methods by several independent criteria, our method has a markedly improved performance in search of module. In addition, our method also shows a strong robustness against the noise in PPI network, which is quite critical for analyzing such a high noise network. More importantly, our model parameter, size k of the clique that is used to measure the density level can use decimal, suggesting that our approach can more precisely detect the module structures in PPI network. We have no doubt that there is still a room for improvement. Indeed, the current method is still far from perfect. Thus, the in-depth works, such as extending the model for analysis of directed and weighted networks with the integration of other high throughput datasets, are required for ADHOC.

## Competing interests

The authors declare that they have no competing interests.

## Authors' contributions

CL and JL conceived of the study and carried out data analysis. YZ participated in the study with useful suggestions. CL, JL and YZ drafted the manuscript. All authors read and approved the final manuscript.
